# Preparation, Characterization, and Analytical Application of Ramipril Membrane-Based Ion-Selective Electrode

**DOI:** 10.1155/2009/954083

**Published:** 2009-04-06

**Authors:** Hassan Arida, Mona Ahmed, Abdallah Ali

**Affiliations:** ^1^Hot Laboratories Center, Atomic Energy Authority, Cairo 13759, Egypt; ^2^Girls College, Ain Shams University, Cairo 11757, Egypt

## Abstract

The fabrication and electrochemical evaluation of two PVC membrane-based Ion-Selective electrodes responsive for ramipril drug have been proposed. The sensitive membranes were prepared using ramipril-phosphomolibdate and ramipril-tetraphenylborate ion-pair complexes as electroactive sensing materials in plasticized PVC support. The electrodes based on these materials provide near-Nernestian response (sensitivity of 53 ± 0.5–54 ± 0.5 mV/concentration decade) covering the concentration range of 1.0 × 10^−2^–1.0 × 10^−5^ mol L^−1^ with a detection limit of 3.0 × 10^−6^–4.0 × 10^−6^ mol L^−1^. The suggested electrodes have been successfully used in the determination of ramipril drug in some pharmaceutical formulations using direct potentiometry with average recovery of >96% and mean standard deviation of <3% (*n* = 5).

## 1. Introduction

**Scheme 1:**
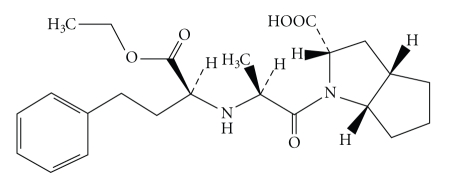
Structure of ramipril.

Ramipril (see Scheme 1) contains not less than 98.0% of C_23_H_32_N_2_O_5_; (2S,3aS,6aS)-1-[(2S)-2-[[(2S)-1-ethoxy-1-oxo-4-phenylbutan-2-yl]amino]propanoyl]-3,3a,4,5,6,6a-hexahydro-2H-cyclopenta[d]pyrrole-2 carboxylic acid. It belongs in a class of drugs called angiotensin converting enzyme (ACE) inhibitors which are used for treating high blood pressure and heart failure and for preventing kidney failure due to high blood pressure and diabetes. ACE is important because it produces the protein, angiotensin II. Angiotensin II contracts the muscles of most arteries in the body, including the heart, thereby narrowing the arteries and elevating the blood pressure. In the kidney, the narrowing caused by angiotensin II also increases blood pressure and decreases the flow of blood. ACE inhibitors such as ramipril lower blood pressure by reducing the production of angiotensin II, thereby relaxing the arterial muscles and enlarging the arteries. The enlargement of the arteries throughout the body reduces the blood pressure against which the heart must pump blood, and it becomes easier for the heart to pump blood. The arteries supplying the heart with blood also enlarge. This increases the flow of blood and oxygen to the heart, and this improves further the ability of the heart to pump blood. The effects of ACE inhibitors are particularly beneficial to people with congestive heart failure. In the kidneys, the enlargement of the arteries also reduces blood pressure and increases blood flow.

Methods in current use for the assay of ramipril, in pharmaceutical preparation, are based on spectrophotometry [1–6], liquid chromatography [7–12], gas chromatography [13, 14], enatioselective biosensors [15], enatioselective membrane [16], voltammetry [17], amperometric biosensor [18], and mass spectrometry [12]. However, most of these methods are sophisticated, tedious, and required many manipulation steps. On the other hand, although, ion-selective electrodes and potentiometric sensors are much simpler, fast, and inexpensive, only one ion-selective electrode has been reported for the determination of ramipril drug as anionic species [19].

In this paper, the preparation, characterization, and analytical application of new two ramipril cationic ion-selective electrodes based on phosphomolibdate and tetraphenylborate-ramipril ion-pair complexes have been reported. The merits offered by the proposed electrodes include the high cationic sensitivity of the drug in the acid media with fast response time (<30 seconds) and long life span (2 months).

## 2. Experimental

### 2.1. Apparatus

The potentiometric measurements were made at 25 ± 1°C, using an Orin (Model A720) digital pH/mV meter and Orion Ross Combination pH electrode (Model 81-02) for all pH measurements. The suggested ramipril PVC membrane-based electrode was used for all potentiometric measurements in conjunction with a double junction reference electrode (Orion Model 90-02) containing KNO_3_ (10% w/v) in the outer compartment. Perkin-Elmer (Norwalk, Conn, USA) 1430 ratio recording IR spectrophotometer was used for structure elucidation of the ion-pair complexes.

### 2.2. Reagents and Materials

All chemicals were of analytical reagent grade unless otherwise stated and doubly distilled water was used throughout. Poly (vinyl chloride) powder (PVC) of high molecular weight (10 000) and dioctyl phthalate plasticizer of purity 98% were obtained from Aldrich chemical company, Inc. (Milwaukee, Wis, USA). Tetrahydrofuran (THF) with a purity of 99% containing 0.025% butylatedhydroxytoluene inhibitor was used as solvent.

Ramipril stock solution (10^−2^ mol L^−1^) was prepared by dissolving 0.4165 g in a minimal volume of acetic acid with continuous stirring and then diluted to 100 mLLwith distilled water. The resulting clear solution of pH 3 was obtained by the addition of small aliquots of 10^−2^ mol L^−1^ HCl. Standard phosphomolibdate and tetraphenylborate solutions (0.2 mol L^−1^) were individually prepared by dissolving appropriate weight of sodium phosphomolibdate and tetraphenylborate, respectively, in a minimum volume of distilled water followed by filtration then completed to 100 mL with distilled water.

### 2.3. The Ramipril Membrane-Based Ion-Selective Electrodes

Ramipril-phosphomolibdate and ramipril-tetraphenylborate ion-pair sensing materials were individually prepared by mixing 30 mL aliquot of 10^−2^ mol L^−1^ ramipril solution with a 30 mL aliquot of 10^−2^ mol L^−1^ aqueous phosphomolibdate and tetraphenylborate solutions, respectively in l00 mL beaker. The obtained precipitates were filtered using a G_4_ sintered glass crucible, washed thoroughly with distilled water, and dried at room temperature. 

Two PVC master membranes based on the suggested sensing materials containing 0.01 g ion-pair complex, 0.350 g dioctylphthalate (DOP), and 0.190 g of poly (vinyl chloride) were individually prepared. Each membrane contents were thoroughly mixed, dissolved in 6 mL aliquot of THF, and transferred to a glass Petri dish (3 cm diameter). The Petri dish was covered with a filter paper and left to stand overnight to allow slow evaporation of the solvent at room temperature. The membranes were sectioned with a cork borer (10 mm diameter) and attached to a length of polyethylene tubing (3 cm length, 8 mm i.d) by using THF. 

A home -made electrode body was used, which consists of a glass tube, to one end of which the poly ethylene tubing was attached and filled with an equimolar mixture of 10^−2^ mol L^−1^ of potassium chloride and ramipril as the internal reference solution. An Ag/AgC1 internal reference wire electrode (1.0 mm diameter) was immersed in the internal solution. This assembly was used in the potentiometric characterization of the electrodes and the subsequent determination of the drug.

### 2.4. Electrochemical Evaluation of the Electrodes

In order to calibrate the suggested electrodes, aliquots (10 mL) of aqueous ramipril solutions (1.0 × 10^−2^–1.0 × 10^−7^ mol L^−1^) were transferred into 50 mL beakers, and the PVC membrane electrode, in conjunction with a double junction Ag/AgCl reference electrode, was immersed in the solution. Alternatively, the drug PVC membrane electrode in conjunction with a double junction Ag/AgC1 reference electrode was immersed in a 50 mL beaker containing a l0 mL aliquot of water. Aliquots (1 mL) of 10^−2^–10^−6^ mol L^−1^ pure drug solution were successively added. The solutions were gently stirred during the measurements and the potential recorded after stabilization to ±0.2 mV and the e.m.f plotted on semilogarithmic paper as a function of the drug concentration. The calibration graphs were used for subsequent determination of unknown concentration of the drug.

The potentiometric selectivity coefficient indicates the extent to which a foreign ion B interferes with the response of the electrode to its primary drug ion A. The potentiometric selectivity coefficients *K*_A, B_^pot^ for the suggested electrodes were measured by separate solutions method SSM [20]. In this method, the potential responses of the electrode in 10^−2^ mol L^−1^ solution of the interferents were measured and recorded. The potential response of the electrode for the drug was separately, obtained in a similar manner at the same concentration level. The selectivity coefficient values were calculated using the simplified form of Eisemnan Nicolsky equation:

(1) $$K_{\text{A},\text{B}}^{\text{pot}}=\frac{E_{1}-E_{2}}{S}+(1-\frac{1}{Z_{2}})\log\;\;a_{1},$$

where *E*_1_ and *E*_2_ are the potential readings of the electrode in separate solutions of the same concentration of the drug and interferants, respectively, *S* is the slope of the drug calibration graph (mV/concentration decade), *a*_1_ is the activity of the drug, and *Z*_2_ is the charge number of the interfering ion.

The effect of pH of the test solution on the potential reading of the suggested drug electrodes was studied by immersing a Ross combination glass electrode (Orion model 81-02), PVC membrane electrode, and a double junction Ag/AgC1 reference electrode in 50 mL beakers containing 30 mL aliquots of 10^−3^ and 10^−4^ mol L^−1^ drug solutions. The pH of each solution was gradually increased and decreased by adding small aliquots of dilute sodium hydroxide and hydrochloric acid, respectively. The potential at each pH value was recorded. The mV-pH profile at each drug concentration was plotted for the two electrode systems.

### 2.5. Analytical Application of the Ramipril Electrodes

In order to investigate the reliability of the proposed electrodes, they have been applied in the determination of the drug using direct potentiometry and potentiometric titration. In the direct potentiometry study, a PVC membrane-based ramipril electrode in conjunction with a double junction reference electrode was immersed in a l0 mL of the appropriate drug solutions of unknown concentration. The potential readings were recorded after stabilization to ±0.2 mV and compared with the calibration graph. The solutions were stirred during measurements, and the electrodes were thoroughly washed with distilled water between measurements.

In the potentiometric titration of ramipril, aliquots (2–6 mL) of 10^−3^ mol L^−1^ of ramipril solution were transferred to 50 mL beakers and diluted to 10 mL with distilled water. The solution was stirred and titrated with a standard 10^−3^ mol L^−1^ sodium tetraphenylborate solution using the suggested ramipril membrane electrode in conjunction with a double junction Ag/AgC1 reference electrode. The electrode potential (*E*) was recorded as a function of the titrant volume (*v*) added, (*E* versus *v*) curves were plotted. The end point was calculated from the maximum slope Δ*E*/Δ*v* versus *v*.

Moreover, different forms and dosages of pharmaceutical preparations were assayed to determine ramipril in different formulations. In this study, aliquot of 5 mL of the drug was transferred to 25 mL beaker containing 15 mL of deionised water, and the pH of the solution was adjusted to pH 3 with a drop of dilute HC1 solution. The solution was then transferred to 25 mL volumetric flask, completed to the mark, shaken well, and transferred to a 100 mL beaker for ISE measurement. For the assay of tablet formulations, 10 tablets were finely powdered, mixed, and an accurate weight equivalent to 0.1% of the tablet was transferred into 50 mL beakers containing 20 mL of deionized water, the pH of the solution was adjusted to pH 3 with a drop of dilute HC1 solution, and transferred to 25 mL volumetric flask, completed with water to the mark, shaken well. The suggested ramipril-based membrane electrode was immersed in conjunction with a double junction Ag/AgC1 reference electrode into the solution. The potential was measured after a stable reading was obtained and compared with that on a calibration graph previously constructed for standard solutions.

## 3. Results and Discussion

### 3.1. The Nature and Composition of Ramipril-Based Membranes

Ramipril—phosphomolibdate and ramipril—tetraphenylborate ion-pair complexes have been prepared and examined as new electroactive ionophores in PVC matrix membranes responsive to ramipril cation. In acid media, ramipril cations readily react with phosphomolibdate and tetraphenylborate anions to form stable water insoluble ion association complexes. The IR studies confirm that the formation of 1:1 ramipril:anion ion-pair association. The potentiometric response characteristics of the proposed electrodes were evaluated, according to IUPAC recommendations [20] using the following electrochemical cell:

$$\begin{array}{c} \text{Ag}/\\ \text{AgCl} \end{array}\;\;|\;\begin{array}{c} {\mathrm{10}}^{-2}\;\text{ mol}\;\;{\text{L}}^{-1}\;\text{ KC1}\\ {\mathrm{10}}^{-2}\;\text{ mol}\;\;{\text{L}}^{-1}\;\text{ ramipril} \end{array}\;\;|\;\begin{array}{c} \text{PVC}\\ \text{membrane} \end{array}\;\;|\;|\;\begin{array}{c} \text{Ramipril}\\ \text{test}\;\text{ solution} \end{array}\;\;|\;|\;\begin{array}{c} \mathrm{10\%}\\ \text{KC1} \end{array}\;\;|\;\begin{array}{c} \text{AgC1}/\\ \text{Ag} \end{array}$$

### 3.2. Electrochemical Evaluation of Ramipril Electrodes

**Figure 1:**
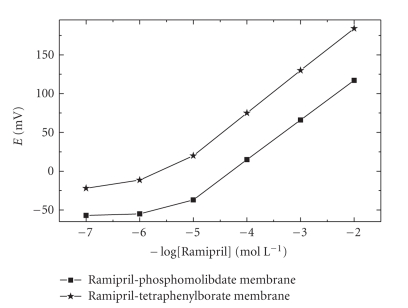
Potentiometric calibration response of ramibril-based selective electrodes.

The potentiometric response characteristics of the investigated ramipril PVC membrane-based electrodes were evaluated from the data collected from four assemblies for each membrane electrode. The results are summarized in (Table 1). The responses of ramipril electrode systems are linear over the concentration range 1 × 10^−2^–1 × 10^−5^ mol L^−1^. The slopes of the calibration plots (Figure 1) are typically 53 ± 0.5 mV and 54 ± 0.5 per concentration decade for ramipril—phosphomolibdate and ramipril—tetraphenyle borate membrane electrode, respectively. Deviation from the ideal Nernstian slope (59.2 mV/concentration decade) stems from the fact that most potentiometric drug sensors respond to the activity of the drug cations rather than the concentration.

**Figure 2:**
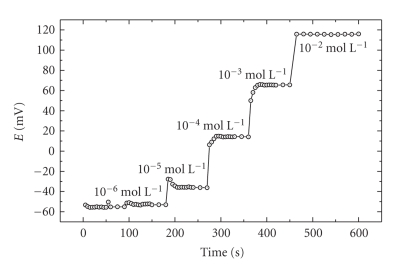
Potentiometric dynamic response of ramipril-phosphomolibdate membrane electrode.

The response time and stability of the membranes have been investigated. In these studies, the time required for ramipril poly (vinyl chloride) membrane electrodes to reach a value of ±1 mV from the final equilibrium potential in the same day after successive immersion in different ramipril solutions was measured. The results obtained (Figure 2) show thats the electrode attain stable potential values within 20 seconds. In addition, the potentials displayed by the electrode in the linear concentration range of ramipril in the same day do not vary by more than ±0.5 mV. The stability of the potential reading for the ramipril-based electrodes is within ±2 mV during the lifetime (2 months) of the electrodes.

**Figure 3:**
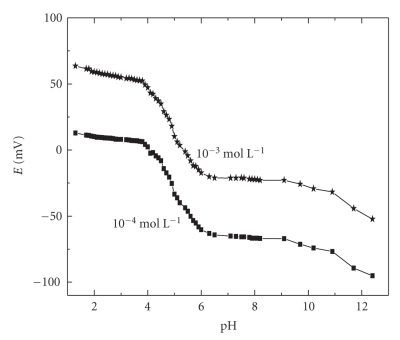
Effect of pH on the potentiometric response of ramipril-phosphomolibdate PVC membrane electrode.

**Figure 4:**
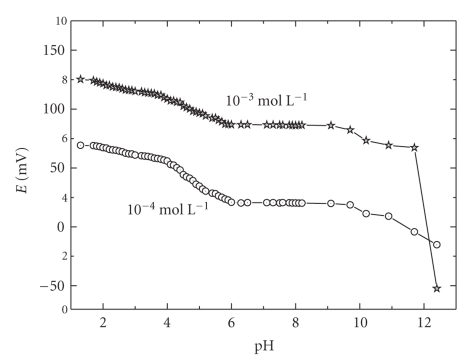
Effect of pH on the potentiometric response of the ramipril-tetraphenylborate PVC membrane electrode.

The effect of pH on the potential readings of the proposed electrode was also examined by recording the e.m.f. of ramipril test solutions (10^−3^ and 10^−4^ mol L^−1^) at various pH values, which were obtained by the addition of very small volumes of hydrochloric acid and/or sodium hydroxide solutions 10^−1^ mol/L of each. The e.m.f. -pH plots presented in Figures 3 and 4 revealed that the potential readings are insensitive to pH changes in the ranges 1.0–3.8 and 1.0–4.0 for the ramipril-phosphomolibdate and ramipeil-tetraphenylborate, respectively. The shape of mV-pH profile depends on the stability of the ion-pair in the membrane as a function of pH, the nature of the drug (protonation or complexation equilibrium) in the test solution, and the sensitivity of the membrane to either [H^+^] or [OH^−^] at low or high pH values, respectively. It was observed that ramipril ion-pair complexes deteriorate in alkaline media causing severe potential shift. At relatively higher pH values, the e.m.f decreased this is probably due to deprotonated species in test solution.

The selectivity coefficients of the investigated electrodes were evaluated using separate solution method (SSM). In this study, the performance of the ramipril electrodes in the presence of some tested organic and inorganic cations was studied. The selectivity coefficient values (*K*_A, B_^pot^) are calculated and presented in Table 2. As can be seen, the electrode offers a reasonable selectivity for the ramipril cation over most of the tested species.

### 3.3. Analytical Application of Ramipril Electrodes

In order to investigate the analytical usefulness of the proposed electrodes, they have been successfully applied in the potentiometer determination of ramipril in aqueous samples as well as in some local pharmaceutical formulations. In the former study, solutions of concentration in the liner range of the tested electrode are prepared from pharmaceutical grade of ramipril and determined by direct potentiometry and potentiometric titration using the proposed ramipril electrodes. The results obtained are summarized in Table 3. In the later study, some local pharmaceutical formulations have been prepared and analyzed by direct potentiometry using the investigated ramipril electrodes. The results obtained are summarized in Table 4. As can be seen, the two electrodes provide a good accuracy (average recovery >96%) and high precision (RSD <3%, *n* = 5) in both studies.

## 4. Conclusion

Two ramipril selective electrodes have been prepared and electrochemicaly evaluated. They provide analytical, useful sensitivity to the drug with fast response time, reliable, and reproducible response. The proposed electrodes have been successfully applied in the determination of ramipril in some pharmaceutical formulations with good accuracy and high precision.

## Figures and Tables

**Table 1:** Response characteristics of ramipril membrane-based selective electrodes.

Parameter	Ramipril	Ramipril
	phosphomolibdate	tetraphenylborate
Slope, mV/decade	53 ± 0.5	54 ± 0.5
Linear range, mol L^−1^	1 × 10^−2^–1 × 10^−5^	1 × 10^−2^–1 × 10^−5^
Lower limit of detection,	4.0 × 10^−6^	3.0 × 10^−6^
mol L^−1^		
Response time, (s)	<20	<15
Life time, (d)	60	50
Working pH range	1–3.8	1–4

**Table 2:** Potentiometric selectivity coefficients (*K*_A, B_^pot^) for the ramipril phosphomolibdate and ramipril-tetraphenylborate selective electrodes.

Interfering species, B	Ramipril-phosphomolibdate	Ramipril-tetraphenylborate
	electrode	electrode
Ramipril	1	1
NH_4_^+^	8.8 × 10^−3^	9.1 × 10^−3^
Co^2+^	8.7 × 10^−3^	8.8 × 10^−3^
Cd^2+^	8.8 × 10^−3^	8.7 × 10^−3^
Ni^2+^	8.8 × 10^−3^	8.7 × 10^−3^
Cr^3+^	4.2 × 10^−2^	4.3 × 10^−2^
Cu^2+^	6.6 × 10^−3^	9.0 × 10^−3^
Ca^2+^	4.6 × 10^−3^	4.8 × 10^−3^
Mg^2+^	9.8 × 10^−3^	9.8 × 10^−3^
Sn^2+^	5.1 × 10^−2^	5.3 × 10^−2^
K^+^	8.8 × 10^−3^	8.6 × 10^−3^
Na^+^	8.1 × 10^−3^	8.5 × 10^−3^
Fe^3+^	8.8 × 10^−3^	8.8 × 10^−3^
Glycine	7.8 × 10^−3^	8.1 × 10^−3^
Phenyl hydrazine	3.2 × 10^−2^	3.2 × 10^−2^
Hydroxylamine	2.2 × 10^−2^	2.7 × 10^−2^

**Table 3:** The accuracy and reliability of result obtained with ramipril PVC matrix membrane electrode.

Sample	Concentration, mol L^−1^	Ramipril-phosphomolibdate		Ramipril-teraphenylborate	
		Found	Recovery %	Found	Recovery %

1	1 × 10^−3^	0.95 × 10^−3^	95.0	0.95 × 10^−3^	95.0
2	2 × 10^−3^	1.87 × 10^−3^	93.5	1.90 × 10^−3^	95.0
3	3 × 10^−3^	2.91 × 10^−3^	97.0	2.92 × 10^−3^	97.3
4	4 × 10^−3^	3.94 × 10^−3^	98.5	3.94 × 10^−3^	98.5
5	5 × 10^−3^	4.98 × 10^−3^	99.6	4.93 × 10^−3^	98.6

Averagere covery			96.7		96.9

**Table 4:** Determination of ramipril in some pharmaceutical preparations.

Sample^(a)^	Nominal content mg/tablet	Ramipril-phosphomolibdate		Ramipril-teraphenylborate	
		Found	Recovery %	Found	Recovery %

1	1.25	1.23	98.4	1.24	99.2
2	2.50	2.43	97.2	2.46	98.4
3	5.0	4.95	99.0	4.95	99.0

Average recovery			98.2		98.8

^(a)^Different pharmaceutical formulations (tritace; aventis).
